# A Case of Functional Para-Aortic Paraganglioma Causing Accelerated Hypertension in an HIV-Positive Patient With Negative 68Ga-DOTATATE PET/CT Findings: Successful Laparoscopic Management

**DOI:** 10.7759/cureus.105340

**Published:** 2026-03-16

**Authors:** Shanmugasundaram Rajaian, Abraham Kurien, Kathirvel Chandramouleeswari, KSNS Udhbhav, Muthu Veeramani

**Affiliations:** 1 Urology, SRM Institutes for Medical Science, Chennai, IND; 2 Pathology and Laboratory Medicine, The Madras Medical Mission, Chennai, IND; 3 Urology, The Madras Medical Mission, Chennai, IND; 4 Urology, SRM Institutes for Medical Science (SIMS) Hospital, Chennai, IND

**Keywords:** 68ga-dotatate, accelerated hypertension, combination antiretroviral therapy, dotatate-negative imaging, extra-adrenal paraganglia, functional paraganglioma, hiv infection, laparoscopic surgery, paraaortic paraganglioma, retroperitoneal space

## Abstract

Functional retroperitoneal paragangliomas are rare neuroendocrine tumors that typically present with catecholamine-mediated hypertension.

68Ga-DOTATATE PET/CT is widely used as a preferred functional imaging modality for the localization of pheochromocytoma and paraganglioma (PPGL), though false-negative results may occur.

The coexistence of paraganglioma and human immunodeficiency virus (HIV) infection is exceptionally rare, with no prior reports of functional retroperitoneal paraganglioma in HIV-positive patients.

We report a 42-year-old female living with HIV on stable antiretroviral therapy who presented with accelerated hypertension. Biochemical evaluation confirmed catecholamine excess with elevated plasma metanephrines. Contrast-enhanced CT demonstrated a 5 × 5 cm left para-aortic mass. Despite strong biochemical and anatomical evidence, 68Ga-DOTATATE PET/CT showed minimal uptake at the lesion site. Following comprehensive preoperative alpha-adrenergic blockade and multidisciplinary optimization, the patient underwent successful laparoscopic excision of the mass. Histopathology confirmed paraganglioma with insulinoma-associated protein 1 (INSM1)-positive staining. Postoperatively, blood pressure normalized, and biochemical markers showed complete resolution. The patient remained disease-free at six-month follow-up with excellent HIV virologic control.

This case represents, to our knowledge, the first reported functional retroperitoneal paraganglioma in an HIV-positive patient. It underscores that DOTATATE-negative imaging should not exclude the diagnosis when biochemical and anatomical evidence is compelling. Laparoscopic resection is feasible and safe in carefully selected patients with well-controlled HIV infection, provided meticulous perioperative preparation and hemodynamic management are implemented.

## Introduction

Pheochromocytomas and paragangliomas (PPGLs) are rare neuroendocrine tumors arising from neural crest-derived chromaffin cells located in the adrenal medulla and extra-adrenal paraganglia. The prevalence of PPGLs in patients with hypertension in general outpatient clinics varies between 0.2 and 0.6%. Although they account for a small proportion of cases of secondary hypertension, the associated morbidity may be significant if unrecognized or untreated [1].

Extra-adrenal retroperitoneal paragangliomas, particularly those located in the para-aortic region, predominantly secrete norepinephrine and are more likely to present with sustained or accelerated hypertension rather than the classic paroxysmal symptom triad described in adrenal pheochromocytoma [2]. Early identification is therefore essential, as timely surgical resection can achieve a cure of hypertension and prevent long-term cardiovascular complications.

Functional imaging plays an increasingly important role in the evaluation of PPGLs. 68Ga-DOTATATE PET/CT targets somatostatin receptor subtype-2 overexpression and has demonstrated high sensitivity for detecting paragangliomas, particularly in extra-adrenal disease [3]. Nevertheless, false-negative DOTATATE studies have been reported. It may be attributable to heterogeneous receptor expression, tumor dedifferentiation, necrosis, or technical factors [4].

Paragangliomas occurring in people living with HIV are exceptionally uncommon, with the existing literature limited to isolated case reports of paragangliomas or other neuroendocrine tumors in HIV-positive individuals. While no causal association has been established, these reports underscore the extreme rarity of this coexistence [5]. Importantly, advances in antiretroviral therapy (ART) have substantially improved perioperative outcomes in well-controlled HIV infection, and current evidence suggests that such patients can safely undergo major surgery with risk profiles comparable to those of HIV-negative individuals [5].

We present what is, to our knowledge, the first reported case of a DOTATATE-negative functional para-aortic paraganglioma causing accelerated hypertension in a female with well-controlled HIV infection, successfully managed with laparoscopic excision. This case highlights potential diagnostic pitfalls when functional imaging findings are discordant and emphasizes that optimally treated HIV infection should not preclude standard oncologic surgical management.

## Case presentation

A 42-year-old female with a nine-year history of HIV infection was referred for evaluation of worsening accelerated hypertension over eight months. She was on a fixed-dose ART regimen of tenofovir alafenamide fumarate 25 mg, emtricitabine 200 mg, and dolutegravir 50 mg with excellent adherence. Her most recent CD4 count was 505 cells/µL, and HIV viral load was undetectable. She had no opportunistic infections or other significant comorbidities.​

Over eight months, she developed progressively worsening headaches, episodic palpitations, and profuse sweating, accompanied by persistently elevated blood pressure levels despite being on amlodipine and metoprolol at maximum doses in a day. On presentation, blood pressure was 228/124 mmHg, and heart rate was 102 beats/min. Fundoscopy demonstrated grade II hypertensive retinopathy changes, and the remainder of the physical examination was unremarkable. Her past medical history was of significance for two cesarean sections nine years ago. She had no neurological deficit.

Given her age and symptom profile, secondary hypertension was suspected and investigated. Her plasma normetanephrines and metanephrines were elevated (Table 1). The rest of the investigations were unremarkable.

**Table 1:** Laboratory investigations.

Parameter	Patient's value	Reference range
Plasma normetanephrine	12.4 nmol/L	<0.50 nmol/L
Plasma metanephrine	2.1 nmol/L	<0.20 nmol/L
Serum creatinine	0.9 mg/dL	0.6-1.2 mg/dL
Serum sodium	138 mmol/L	135-145 mmol/L
Serum potassium	4.2 mmol/L	3.5-5.0 mmol/L
Fasting blood glucose	90 mg/dL	70-100 mg/dL
Thyroid-stimulating hormone	2.3 µIU/mL	0.4-4.0 µIU/mL
CD4 count	505 cells/µL	500-1500 cells/µL
HIV viral load	Undetectable	Undetectable

**Figure 1:**
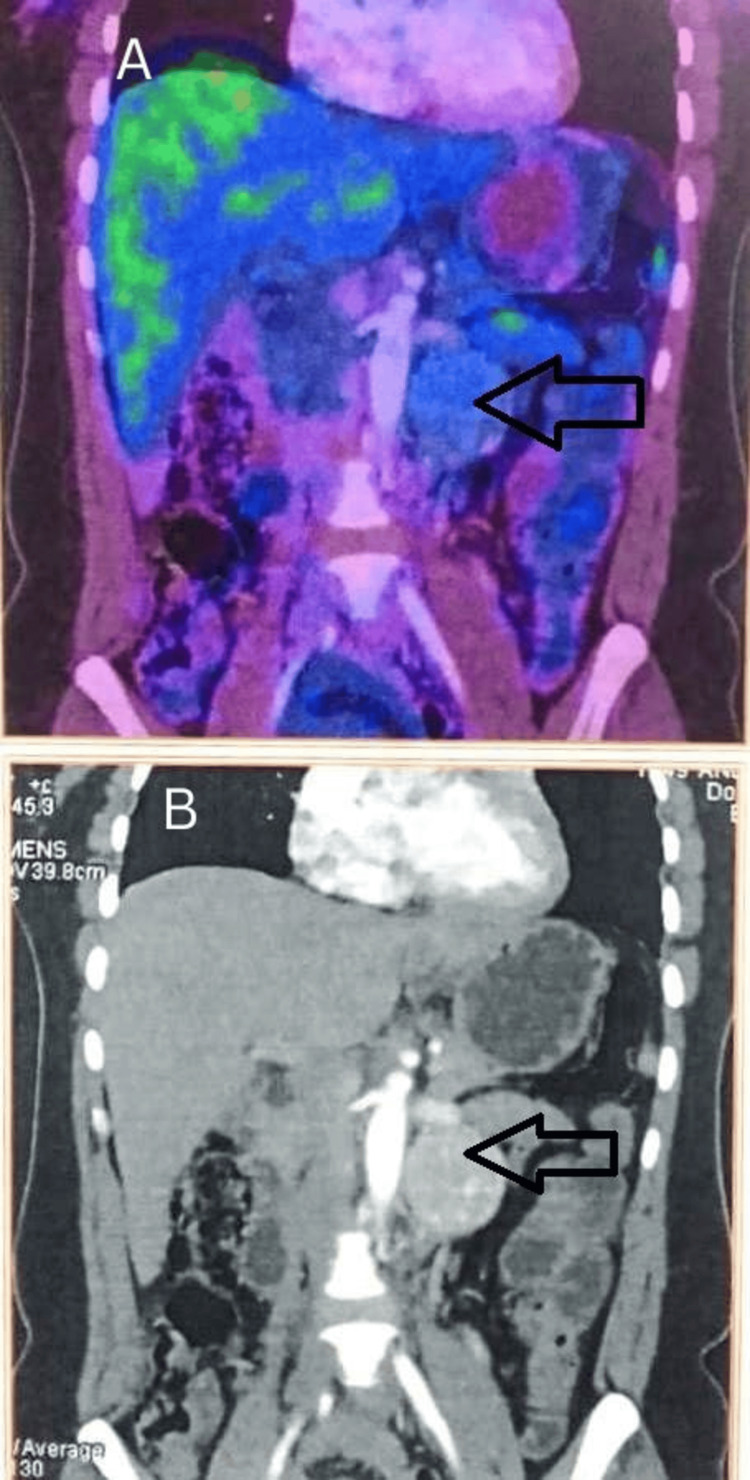
68Ga-DOTATATE PET/CT & contrast-enhanced CT of the abdomen. Panel A: Coronal section of 68Ga DOTATATE PET/CT shows very minimal uptake of the tracer by the lesion (black hollow arrow) in spite of very good contrast enhancement of the lesion located below the level of the left renal vein and lateral to the aorta in panel B (black hollow arrow).

Contrast-enhanced CT of the abdomen showed a well-defined, avidly enhancing 5 × 5 cm soft-tissue mass in the left para-aortic region. 68Ga-DOTATATE PET/CT demonstrated only faint uptake (Figure 1). The superior limit of the swelling was the left renal vein. These features were suggestive of a retroperitoneal paraganglioma.​

68Ga-DOTATATE PET/CT was performed for functional characterization and staging. Unexpectedly, the known para-aortic mass demonstrated only faint DOTATATE uptake, with a lesion-to-liver ratio well below thresholds usually regarded as positive, and no other abnormal foci were identified (Figure 1). Despite this discordant functional imaging, the combination of markedly elevated metanephrines and characteristic CT findings favored a diagnosis of functional paraganglioma [3].​

The patient was prepared for surgery with phenoxybenzamine 60 mg twice per day, gradually titrated over approximately two weeks to achieve seated blood pressure <130/80 mmHg with mild orthostatic symptoms, along with liberal salt and fluid intake. A beta-blocker (propranolol 40 mg) was added after adequate alpha-blockade to control tachycardia, according to standard perioperative protocols for PPGL. ART was continued unchanged after review, excluding major interactions with planned anesthetic and vasoactive agents.

**Figure 2:**
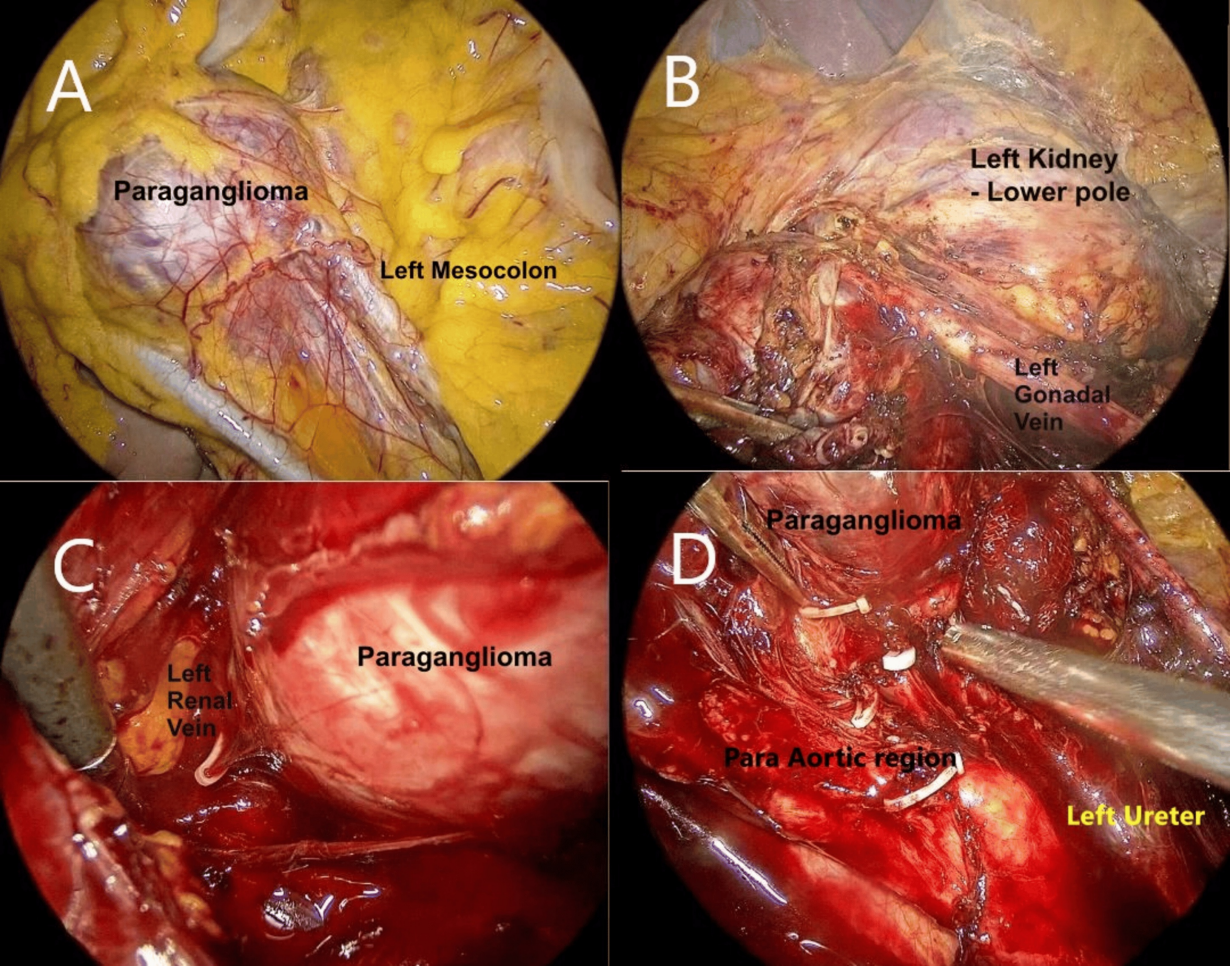
Laparoscopic excision of the paraganglioma. Panel A:Diagnostic laparoscopic view showing the paraganglioma through the left mesocolon, splaying the left gonadal vein and the mesocolic vessels. Panel B: After reflection of the left mesocolon, the left gonadal vein was isolated, and a vein draining from the mass was dissected. Panel C: A venous tributary draining into the left renal vein was clipped early during mobilization of the paraganglioma mass, preventing a surge of catecholamines into circulation. Panel D: The feeding vessels to the mass from the para-aortic region were clipped and divided.

She underwent laparoscopic transperitoneal excision of the para-aortic mass (Figure 2). In the right lateral decubitus position, a three-port configuration was used. After mobilizing the left colon and exposing the retroperitoneum, a well-defined vascular mass was identified adjacent to the left gonadal and renal vein and also close to the para-aortic region. Dissection was performed along the periphery of the lesion with minimal manipulation of the tumor. The veins that drained the lesion to the renal vein and main tributary to the stretched gonadal vein were clipped early and divided. The transient intraoperative hypertensive surges were managed with short-acting vasodilators, and brief hypotension after tumor vein ligation responded to volume and low-dose vasopressin infusion support. Operative time was 2.5 hours, and blood loss was 100 ml. The mass was removed intact in a retrieval bag, adopting all the universal precautions by expanding the lowermost working port.

**Figure 3:**
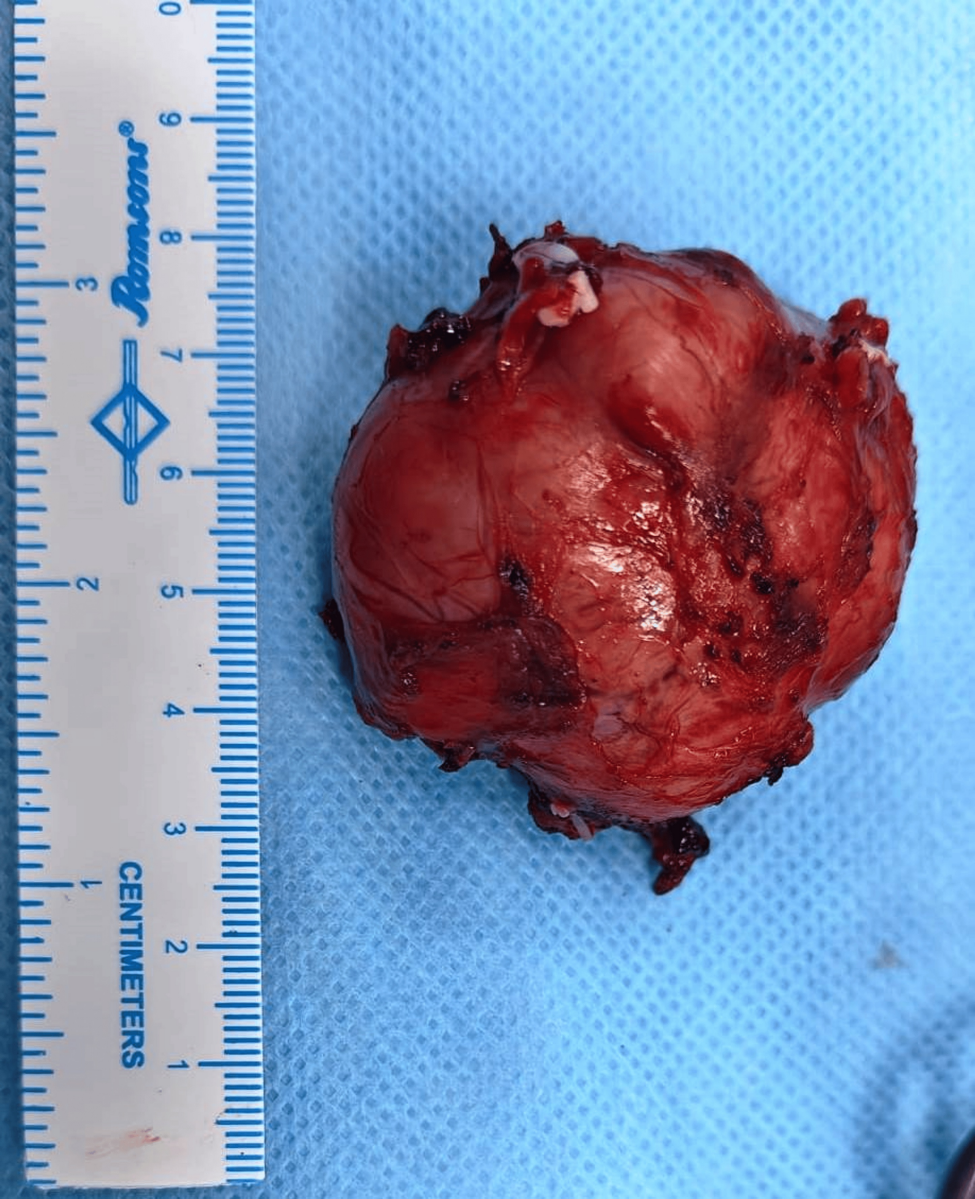
Specimen of the excised left para-aortic mass. A well-encapsulated tan brown swelling with multiple vascular arterial feeders was excised after securing with hemostatic clips.

**Figure 4:**
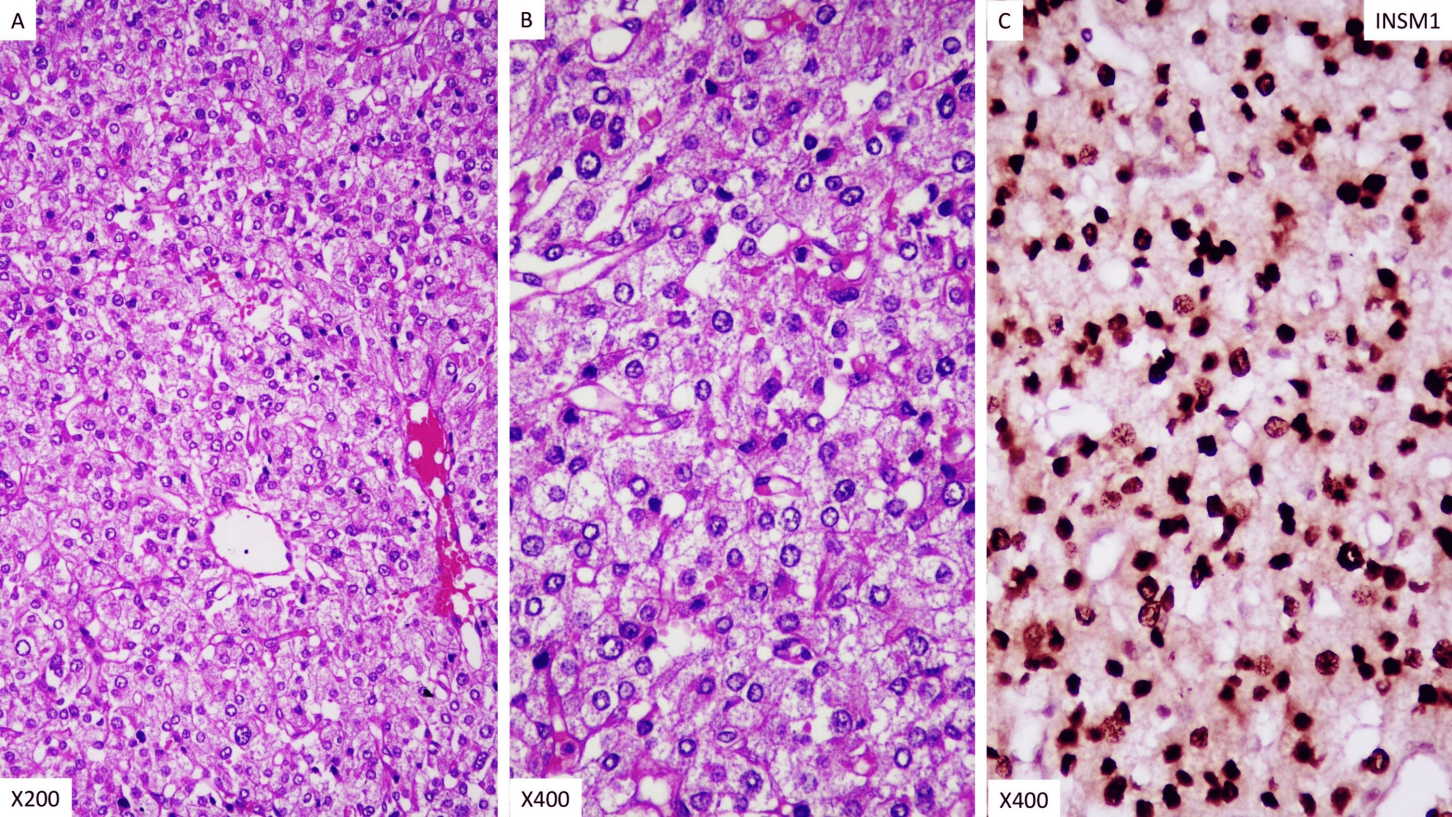
Histopathology and immunohistochemistry of the excised mass. Panel A: Hematoxylin & eosin stain (200x) showing polygonal tumor cells arranged in islands and nests (zellballen pattern) with prominent vascular channels. Panel B: Hematoxylin & eosin stain (400x) showing well-arranged tumor cells with fine reticular cytoplasm and vesicular nuclei. The nucleoli were not prominent. Panel C: Immunohistochemistry staining with insulinoma-associated protein 1 (INSM1) showing positive staining.

Grossly, the tumor was a well-encapsulated tan-brown spherical mass measuring 5 x 5 cm (Figure 3). Microscopy showed classical zellballen architecture with nests of uniform polygonal cells separated by delicate vascular stroma, low mitotic activity, and no necrosis or vascular invasion (Figures 4A, 4B). Immunohistochemistry demonstrated nuclear positivity for insulinoma-associated protein 1 (INSM1) (Figure 4C) [6], a sensitive neuroendocrine marker supporting the diagnosis of paraganglioma.

Postoperatively, the patient’s blood pressure became normal, and all antihypertensive medications were discontinued on the next postoperative day. She was discharged on postoperative day three after thorough recovery. At six weeks, plasma metanephrines had all returned to normal ranges. At six months, she remained normotensive without medications, free of symptoms, and biochemically disease-free. Her CD4 count remained at 550 cells/µL with persistently undetectable viral load, indicating that surgery did not compromise HIV control.

## Discussion

This case underscores several important points: the limitations of DOTATATE imaging in PPGL, the feasibility of laparoscopic resection of functional retroperitoneal paragangliomas, and the safety of major endocrine surgery in patients with well-controlled HIV.

DOTATATE-negative functional paraganglioma

68Ga-DOTATATE PET/CT is now considered one of the most sensitive tools for localizing PPGL, with lesion-based sensitivities often exceeding 95% and clear superiority over conventional metaiodobenzylguanidine (MIBG) scintigraphy [3]. False-negative DOTATATE studies may occur due to heterogeneous receptor expression, tumor dedifferentiation, or necrosis [3,4].

In our patient, DOTATATE uptake in the paraaortic mass was below the liver background despite unequivocal biochemical evidence of catecholamine excess and typical CT appearance and location. Histology confirmed a well-differentiated tumor. Focal necrosis and possible receptor heterogeneity may have contributed to low tracer uptake [3]. The key clinical lesson is that a negative DOTATATE study does not rule out functional paraganglioma when biochemical and anatomical data are strongly supportive [1,2]. In such situations, management decisions should prioritize biochemistry and cross-sectional imaging over a single functional modality.

Laparoscopic management of retroperitoneal paraganglioma

Complete surgical excision remains the only curative treatment for localized PPGL. Laparoscopic and retroperitoneoscopic approaches are increasingly used for suitable adrenal and extra-adrenal lesions, offering reduced blood loss, shorter hospital stay, and faster recovery without compromising oncologic outcomes [7]. A series of retroperitoneal paragangliomas treated laparoscopically reported high rates of complete resection, low complication rates, and excellent medium-term oncologic control in carefully selected patients [7-9].

Key requirements for safe laparoscopic resection include moderate tumor size, absence of overt invasion or major vascular encasement, meticulous preoperative alpha-blockade, and close intraoperative hemodynamic monitoring. Our patient fulfilled these criteria, and her perioperative course mirrors outcomes reported in contemporary series, supporting minimally invasive surgery as an appropriate strategy for selected functional para-aortic paragangliomas in experienced centers.

HIV infection and surgical outcomes

Neuroendocrine tumors are rare in people with HIV. Published data include sporadic reports of paraganglioma or other neuroendocrine tumors in HIV-positive patients, suggesting no strong causal relationship but highlighting extreme rarity [5]. To the best of our knowledge, a functional retroperitoneal paraganglioma in a patient with HIV has not been previously reported.

Modern guidelines for perioperative care in adults with HIV emphasize that patients with CD4 counts above 200 cells/µL and suppressed viral load do not have substantially increased surgical risk compared with HIV-negative individuals [10] and should generally be managed according to standard surgical principles. Continuation of ART wherever possible, careful review of drug-drug interactions, and appropriate infection prophylaxis based on immune status are central recommendations. Our patient, with a CD4 count above 500 cells/µL and an undetectable viral load, underwent major laparoscopic endocrine surgery without complications.

Patients with well-controlled HIV infection can safely undergo major surgery with perioperative risk comparable to HIV-negative individuals [10].

The prevalence of PPGLs in individuals carrying a germline mutation in PPGL susceptibility genes may be around 50%. Patients with hereditary PPGLs typically present with multifocal disease and at a younger age than those with sporadic neoplasms [11]. We have deferred the germline mutation testing in view of her age and unifocality of the disease.

## Conclusions

This case describes a DOTATATE-negative functional para-aortic paraganglioma causing accelerated hypertension in a female with well-controlled HIV infection, successfully treated by laparoscopic excision. Negative somatostatin receptor PET may not exclude PPGL when biochemical and anatomical evidence is compelling. Minimally invasive surgery can be safely performed in carefully optimized HIV-positive patients. Clinicians should integrate biochemical testing, cross-sectional imaging, and multidisciplinary planning rather than relying solely on a single functional imaging modality when managing suspected paraganglioma.
